# Supplementary material for: DEVOUR: Deleterious Variants on Uncovered Regions in Whole-Exome Sequencing
Source: PeerJ. 2023 Sep 15;11:e16026. doi: 10.7717/peerj.16026 (PMC10506587; doi:10.7717/peerj.16026)

**Supporting Information**

**DEVOUR:** Deleterious Variants on Uncovered Regions

Erdem Türk, Ayhan Yüksek, Akif Ayaz and Barış Ethem Süzek

Data Preparation: We downloaded one of the WES samples (NCBI SRA: [ERR1840777](https://www.ncbi.nlm.nih.gov/sra/?term=ERR1840777)) of the WES project for Hirschsprung disease (NCBI Bioproject: [PRJEB19327](https://www.ncbi.nlm.nih.gov/bioproject/?term=PRJEB19327)) from NCBI SRA and aligned it to the reference genome (build 19). We then fed the resulting alignment file to DEVOUR along with the exome capture kit ([TruSight_One_v1.1.bed](https://support.illumina.com/content/dam/illumina-support/documents/downloads/productfiles/trusight/trusight-one-file-for-ucsc-browser-v1-1.zip)) used by the Illumina sequencing platform.

An example run for a WES sample: The following screenshots illustrate how DEVOUR is used to analyze a WES sample step by step.

**Figure 1.** The graphical user interface for the first step of the analysis. The first step aims to identify potentially uncovered or low coverage genomic regions in a WES experiment. A read alignment file in BAM or SAM format and an exome capture file for the WES kit used in BED format are provided as input files. A read depth threshold in the range 0 (no coverage regions) to 15 and the human reference genome release name are also set as input.


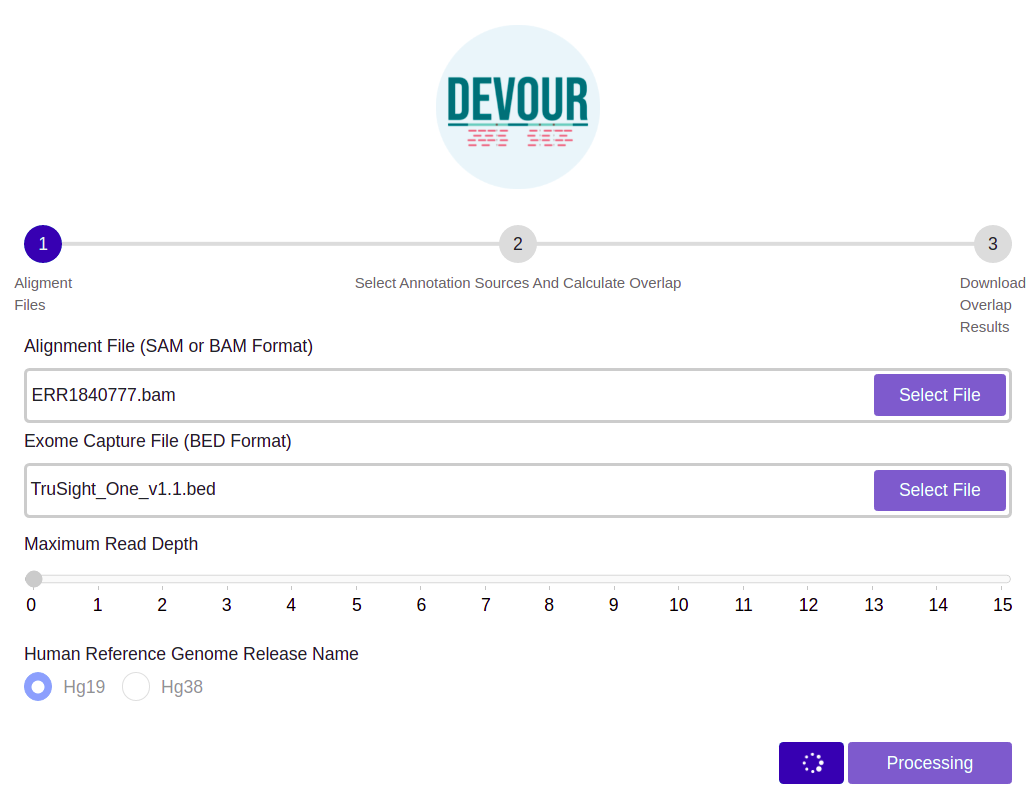


**Figure 2.** The graphical user interface for the second step of the analysis. The second step aims to enrich the low coverage regions with variant annotations as a step toward assessing the clinical significance of variants found on these regions. Variant annotation sources are selected from the list of custom and ANNOVAR-derived configured in the local instance of DEVOUR.


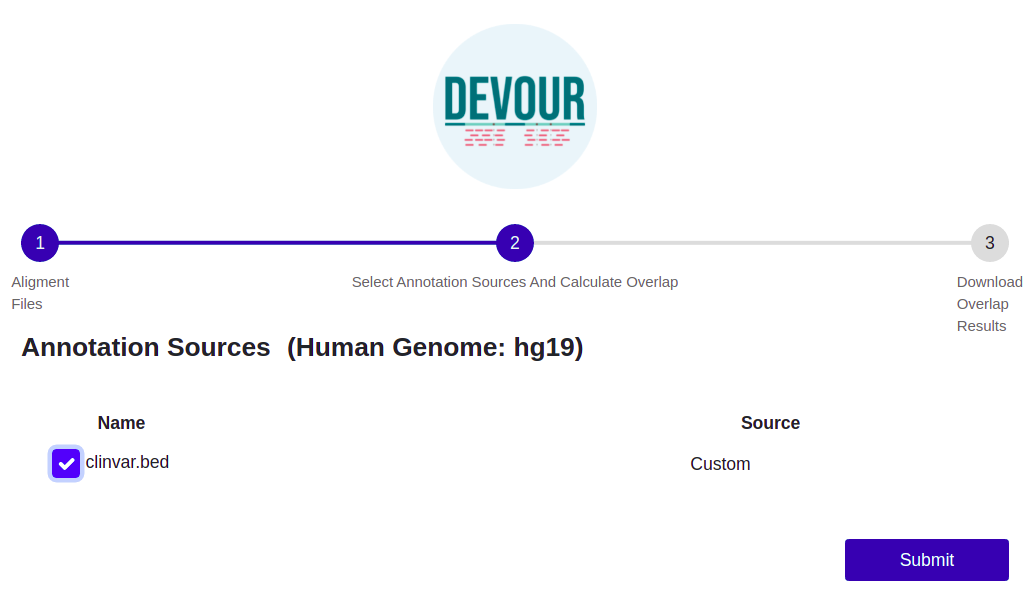


**Figure 3.** The graphical user interface for the final step of the analysis. The final step aims to provide files to assist clinical diagnosis highlighting the variants on potentially uncovered or low coverage genomic regions with clinical significance (e.g., pathogenicity). Users can export the annotations of variants located on potentially uncovered or low coverage genomic regions in TSV or Excel format.


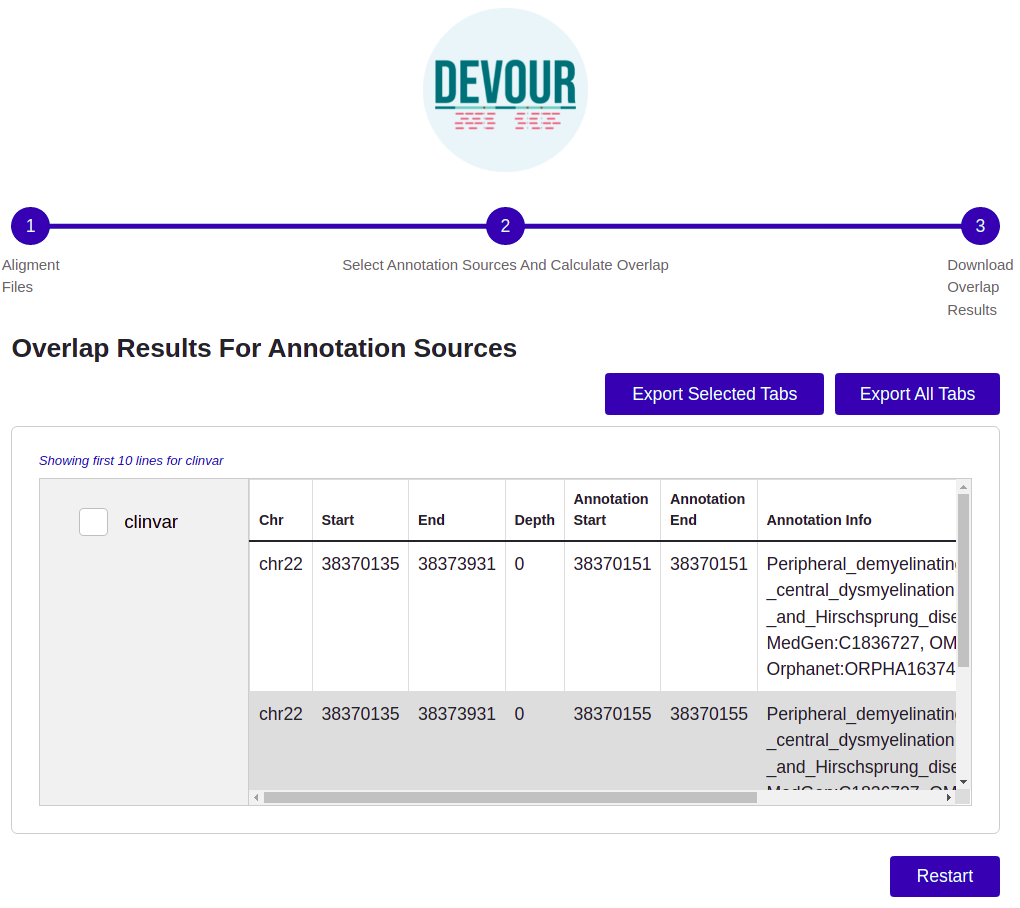

Supplement: Supplemental Information 1 — An example run for the SRA sample ERR1840777. [file peerj-11-16026-s001.docx]
